# *MoGLN2* Is Important for Vegetative Growth, Conidiogenesis, Maintenance of Cell Wall Integrity and Pathogenesis of *Magnaporthe oryzae*

**DOI:** 10.3390/jof7060463

**Published:** 2021-06-08

**Authors:** Osakina Aron, Min Wang, Lianyu Lin, Wajjiha Batool, Birong Lin, Ammarah Shabbir, Zonghua Wang, Wei Tang

**Affiliations:** 1 Fujian Universities Key Laboratory for Plant-Microbe Interaction, College of Plant Protection, Fujian Agriculture and Forestry University, Fuzhou 350002, China; osakina.aron@yahoo.com (O.A.); lianyully@163.com (L.L.); jiaali174@yahoo.com (W.B.); ammara.shabbir@yahoo.com (A.S.); 2 State Key Laboratory of Ecological Pest Control for Fujian and Taiwan Crops, Fujian Agriculture and Forestry University, Fuzhou 350002, China; 17750243431@139.com (M.W.); lin18105028780@163.com (B.L.); 3 Marine and Agricultural Biotechnology Center, Institute of Oceanography, Minjiang University, Fuzhou 350108, China

**Keywords:** glutamine, pathogenicity, glutamine synthetase, *Magnaporthe oryzae*, cell wall integrity

## Abstract

Glutamine is a non-essential amino acid that acts as a principal source of nitrogen and nucleic acid biosynthesis in living organisms. In *Saccharomyces cerevisiae*, glutamine synthetase catalyzes the synthesis of glutamine. To determine the role of glutamine synthetase in the development and pathogenicity of plant fungal pathogens, we used *S. cerevisiae* Gln1 amino acid sequence to identify its orthologs in *Magnaporthe oryzae* and named them MoGln1, MoGln2, and MoGln3. Deletion of *MoGLN1* and *MoGLN3* showed that they are not involved in the development and pathogenesis of *M. oryzae*. Conversely, Δ*Mogln2* was reduced in vegetative growth, experienced attenuated growth on Minimal Medium (MM), and exhibited hyphal autolysis on oatmeal and straw decoction and corn media. Exogenous l-glutamine rescued the growth of Δ*Mogln2* on MM. The Δ*Mogln2* mutant failed to produce spores and was nonpathogenic on barley leaves, as it was unable to form an appressorium-like structure from its hyphal tips. Furthermore, deletion of *MoGLN2* altered the fungal cell wall integrity, with the Δ*Mogln2* mutant being hypersensitive to H_2_O_2_. MoGln1, MoGln2, and MoGln3 are located in the cytoplasm. Taken together, our results shows that *MoGLN2* is important for vegetative growth, conidiation, appressorium formation, maintenance of cell wall integrity, oxidative stress tolerance and pathogenesis of *M. oryzae*.

## 1. Introduction

Glutamine is a non-essential amino acid and is required in a vast number of metabolic pathways in living organisms. For example, in humans glutamine is required in pathways such as nitrogen metabolism, ammonia detoxification, acid–base homeostasis, osmotic regulation, cell signaling, and proliferation [1,2]. In addition, it has been reported that glutamine acts as a precursor for neurotransmitters and a substrate for immune cells [3,4]. Furthermore, it has been shown that glutamine is used for the synthesis of biomolecules such as glucose, purines, pyrimidines, adenosine monophosphate, and nicotinamide adenine dinucleotide (NAD+) [5,6,7]. Since glutamine is a crucial metabolite in the metabolism of nitrogen, the intracellular glutamine levels are tightly regulated. Experimental data for various fungi have provided evidence that glutamine is a key effector of nitrogen catabolite repression (NCR), a regulatory cascade that is biased toward or prefers the utilization of reduced nitrogen sources such as ammonium and glutamine at the expense of more complex and energy-demanding ones, e.g., nitrate, purines, and proteins [8,9]. A classic example occurs in *Saccharomyces cerevisiae*, where a well-established model of the target of rapamycin (Tor) exists, where intracellular glutamine levels are sensed by the (Tor) complex kinase 1 (TorC1), thus relaying the signal of glutamine availability to the GATA-type transcription factors Gln3 and Gat1. These GATA factors then respond by activating the transcription of NCR-sensitive genes during nitrogen-starvation conditions or when non-preferred nitrogen sources are present [10,11]. Glutamine signals for nitrogen metabolite repression can also be sensed from ammonia, glutamate, and nitrate [12,13,14].

Glutamine synthetase (GS) catalyzes the biosynthesis of glutamine, which acts as a principal nitrogen source for the synthesis of nucleic acid and protein synthesis. In living cells, ammonium assimilation occurs in two main ways [15]: NADP-dependent glutamate synthesis, a reaction catalyzed by glutamate dehydrogenases, in which ammonium and 2-oxoglutarate act as the substrates [16]; and the assimilation of ammonium by the glutamine synthetase, an ATP-dependent reaction that results into the formation of an intermediate product γ-glutamyl phosphate from glutamate, which eventually combines with ammonia to generate glutamine and inorganic phosphate [15,16]. Several researchers have studied the biological role, physico-chemical properties, and kinetic properties of GS from different sources [17]. Methionine sulfoximine (MetSox) and phosphinothricin (PPT) have been reported to be the inhibitors of GS activity, as they tightly bind to its active site of GS [16]. Because of the inhibition property of these two molecules on GS, GS can act as an important target for bio-pesticides to be used in the agricultural industry. GS exhibits both the biosynthetic and γ-glutamyl transferase activities, with these two different forms based on whether the GS is in adenylylated or non-adenylylated forms [18,19]. The biosynthetic activity catalyzes the formation of glutamine from glutamate and ammonia, whereas the γ-glutamyl transfer activity catalyzes the transfer of γ-glutamyl moieties to peptides, amino acids, or water [20]. GS is classified as GSI, GSII, or GSIII [21]. GSI enzymes were thought to exist exclusively in prokaryotes, and their structures were shown to be dodecameric [22,23]. Later, GSI enzymes were also identified in mammals and plants [24,25]. In filamentous fungi, GSII family proteins were identified, and in most cases, as one GS-encoding gene present in the fungal genomes. However, two different subunits of GSα and GSβ were found to encode the GSII family in the filamentous fungus *Neurospora crassa* [26,27]. The presence of these two GS encoding genes in *N. crassa* was confirmed by genome sequencing. Since GS plays a crucial role in glutamine biosynthesis and nitrogen regulation. The activity of GS is tightly regulated to a level that allows the amount of glutamine available for various metabolic pathways to be strictly controlled. Studies on how GS is regulated have clearly been shown in Prokaryotes. For instance, in *Escherichia coli*, glutamine and end products of glutamine metabolism, such as AMP, ADP, and other nucleotides, offer a cumulative feedback inhibition to GS1 by competing with the substrate glutamate for the active site [28]. In *Bacillus subtilis*, GSI activity is feedback-inhibited by glutamine [29] and this inhibited form of GS controls the DNA-binding capabilities of the TnrA and GlnR transcription factors via protein interaction [29,30]. Both these transcription factors eventually regulate gene expression in response to changes in nitrogen availability.

Because glutamine is the major substrate for numerous metabolic pathways, it is an important amino acid for normal functioning of living organisms; therefore, deficiency of glutamine caused by a defect in GS interferes with normal life. In *Drosophila melanogaster*, mutations in the gene encoding the mitochondrial glutamine synthetase I (GSI) resulted in embryo lethality, consequently leading to female sterility [31], Moreover, in mice, GS was also found to be essential in early embryogenesis [31]. In humans, mutations in the GLUL gene (OMIM: 138290), which encodes for GS, were reported to cause an ultra-rare recessive inborn error of metabolism—congenital glutamine synthetase deficiency [32], whereas in plants GS has been reported to be essential for normal plant growth and development [33]. In filamentous fungi such *Aspergillus nidulans* and *Schizosaccharomyces pombe,* GS plays a crucial role in growth and development [34,35], while deletion of the GS in plant pathogenic fungi *Gibberella fujikuroi* GS gene showed that, besides influencing growth, GS has a significant impact on the transcriptional control of primary and secondary metabolism [36].

*Magnaporthe oryzae* is a devastating hemibiotrophic fungus that attacks most cereals, including rice, thus posing a great challenge to global food security. Due to its economic significance and genetic tractability, the blast fungus has been developed as a model organism for plant-fungus interaction studies [37]. Infections begin when conidia germinate and develop a specialized dome-shaped structure called an appressorium upon coming in contact with the rice plant surface [38]. The mature appressorium then accumulates enormous turgor pressure (8 MPa), which helps it puncture the rice cuticle, thus facilitating its entry into plant cells [39]. While inside the host cell, the fungus differentiates into bulbous invasive hyphae (IH), which eventually colonizes the adjacent plant cells. Rice blast fungus initially adopts a hemibiotrophic infection strategy, which lasts approximately four to six days; the fungus colonizes the living host cells without causing damage to the host at this stage. Later it enters into a devastating necrotrophic phase, where the fungus rapidly destroys the infected host tissue [40].

Previously, findings have documented the role of GS in growth and development in different organisms [33,35,41]. However, the exact influence of GS in the development and pathogenicity of plant fungal pathogens is unclear. In this study, we identified three genes that encode GS in rice blast fungus. We established that *MoGLN1* and *MoGLN3* had no influence on the development and pathogenicity of *M. oryzae.* In contrast, our findings showed that *MoGLN2* is important in glutamine biosynthesis, exerting a significant effect on vegetative growth, conidiogenesis, appressorium-like structure formation, and melanin biosynthesis, and that it was crucial for the maintenance of cell wall integrity and oxidative stress tolerance in *M. oryzae.* Our findings, therefore, suggest that glutamine biosynthesis mediated by *MoGLN2* could provide a suitable target point for antifungal design against plant fungal pathogens.

## 2. Materials and Methods

### 2.1. Fungal Strains and Culture Conditions

The wild-type (WT) Guy11 and mutant strains of *M. oryzae* were cultured at 25 °C using complete media (CM: 0.6% yeast extract, 0.6% casein hydrolysate, 1% sucrose, 1.5% agar) as described in [42]. Other media used in this study include minimal media (MM: 6 g of NaNO_3_, 0.52 g of KCl, 0.52 g of MgSO_4_, 1.52 g of KH_2_PO_4_, 10 g of glucose, and 15 g of agar in 1 L of double-distilled water), straw decoction and corn media (SDC: 100 g of rice straw, 40 g of corn flour, and 15 g of agar in 1 L of double-distilled water), and oatmeal agar media (OTM: 50 g of oatmeal and 15 g of agar in 1 L of double-distilled water). Samples for genomic DNA extraction, total RNA, and protoplast preparation were cultured in liquid CM in an orbital shaker at 110 rpm for 3 days.

Sensitivity assays were performed by culturing strains on CM plates supplemented with cell wall enforcing agents (0.01% SDS, 200 μg/mL Congo red, 200 μg/mL Calcofluor white), oxidative stress agent 2.5 mM, and 5 mM hydrogen peroxide (H_2_O_2_) for 8 days at 28 °C inside a dark chamber.

To induce conidiation, strains were cultured on rice bran agar (2% rice bran, 1.5% agar; pH 6.5) for 10 days at 28 °C in the dark followed by 3 days of continuous light illumination. Conidia were collected in 5 mL of distilled water, filtered using three-layer lens paper, and counted with a hemocytometer under a light microscope.

### 2.2. Target Gene Deletion and Complementation in M. oryzae

To generate *MoGLN* deletion mutants, the split-marker approach [43] was adopted in the construction knockout vectors used for deleting each of the *MoGLN* genes in *M. oryzae*. The upstream and downstream flanking fragments of *MoGLN* genes were amplified with the primer pairs listed in Table S1. The amplified PCR fragments were ligated with hygromycin phosphotransferase (hph) cassette fragments amplified with primers HYG/F + HY/R and YG/R + HYG/R (Table S1) by overlapping PCR.

Protoplast preparation and transformation procedures were performed as previously described [44] Transformants were selected on TB3 medium supplemented 250 μg/mL hygromycin B (Roche Applied Science, Penzberg, Germany) and 200 μg/mL G418 (Invitrogen, Carlsbad, CA, USA), and the mutants were verified by Southern blotting analysis.

To generate complementation strains, fragments containing the full length of *MoGLN1, MoGLN2,* and *MoGLN3* genes and their respective 2.3 Kb native promoters were amplified with primer pairs Gln1 com F/R, Gln2com F/R, and Gln3 comF/R (Table S1). The resulting PCR products were cloned in pKNTG vector containing neomycin resistance. Each construct was transformed in its respective mutant protoplast.

### 2.3. Appressorium Formation, Penetration, and Infection Assays

Conidia collected from 10-day-old rice-bran culture were adjusted to (5 × 10^4^ spores/mL) using sterilized double-distilled water with 0.02% (*v*/*v*) Tween-20 solution. Appressorium formation assays were performed by adding 20 µL of conidial suspension from Guy11; Δ*Mogln1* and Δ*Mogln3* strains were on an artificial hydrophobic coverslip and incubated in darkness at 28 °C. Appressorium formation was then examined at 4 h, 8 h, 12 h, and 24 h time intervals. Conidia germination and appressorium formation on inductive surfaces were measured as described previously [45].

To observe the formation of the appressorium-like structure, mycelial plugs from Guy11, Δ*Mogln1,* Δ*Mogln2,* and Δ*Mogln3* strains were inoculated on artificial cover slips and 10-day-old barley leaves; appressorium-like structure formation was then observed after 30 h.

Rice infection was performed by spraying 3-week-old rice (*Oryzae sativa* cv. CO39) seedlings with the Guy11, Δ*Mogln1,* and Δ*Mogln3* strain conidial suspension (5 × 10^4^ spores/mL). The infected plants were incubated in a humid chamber at 28 °C for 24 h in darkness and later transferred to a 12 h photoperiod chamber. Leaves were then imaged 7 days after infection.

For the barley infection assay, mycelial plugs derived from Guy11, Δ*Mogln1,* Δ*Mogln2,* and Δ*Mogln3* were incubated on 10-day-old barley leaves at 28 °C for 24 h in darkness. Later, they were transferred to light conditions and imaged after 7 days.

To observe penetration and invasive hyphal growth, 10 µL of Guy11, Δ*Mogln1,* and Δ*Mogln3* conidial suspension (5 × 10^4^ spores/mL) was repeatedly dropped on 10-day-old barley leaves. The infected leaves were then incubated for 30 h, 48 h, and 72 h at 28 °C under humid conditions; penetration and invasive hyphal growth were examined using a microscope.

### 2.4. Nucleic Acid Manipulation, Southern Blotting Analysis, and qRT-PCR

DNA extraction was performed using cetyltrimethylammonium bromide (CTAB) [46], gel electrophoresis, enzyme digestion, and ligation, and Southern blot hybridization was performed using standard procedures [47]. Probe labelling, hybridization, and detection were performed with a DIG High Prime DNA Labeling and Detection Starter Kit (Roche Applied Science, Penzberg, Germany).

Total RNA was isolated from frozen fungal mycelia and rice leaves using a Magen universal RNA kit as previously described [48]. To measure the relative abundance of gene transcripts, RNA was extracted from mycelia cultured in CM liquid medium for 3 days at 28 °C in an orbital shaker (110 rpm). To measure the relative abundance of *MoGLN*, *MoGLN2,* and *MoGLN3* transcripts during fungal developmental stages, the total RNA samples were extracted from mycelia grown in CM liquid medium, conidia, and rice leaves inoculated with the conidia of Guy11 (1 × 10^8^ spores/mL) for 8, 24, 48 and 72 h.

Total RNA for all the samples was extracted using a Magen universal RNA kit, as described previously [48]. For RT-PCR and quantitative real time RT-PCR (qRT-PCR), 5 mg of total RNA was converted to cDNA using HiScript^®^11Q select RT supermix (vazyme). The qRT-PCR data were generated with an Eppendorf Realplex2 master cycler (Eppendorf AG 223341, Hamburg, Germany). Thermocycler conditions were as follows: 2 min at 95 °C, followed by 40 cycles of 95 °C for 15 s, 60 °C for 30 s, and lastly, the melting curve stage of 95 °C for 15 s, 60 °C for 15 s, and 95 °C for 15 s. The stable expression actin gene (MGG_03982) was used as internal control, and three independent replicates were performed for each experiment; the qRT-PCR primers used are listed in Table S1. Data analysis was performed using the delta delta-CT (2−ΔΔCT) method, as described in [49].

### 2.5. Western Blot Assays

The wild-type Guy11 and the mutant strains were grown in liquid CM medium at 28 °C for 4 days with agitation at 110 rpm. Total protein was extracted from mycelia as described previously [50]. The intensity of the signal corresponding to phosphorylated Mps1 was detected by binding of phospho-p44/42 MAPK (ERK1/2) (Thr202/Tyr204) (D13.14.4E) Rabbit mAb and p44/42 MAPK (ERK1/2) antibodies (Cell Signaling Technology, Beverly, MA, USA).

### 2.6. RNA Isolation, Library Construction, and Sequencing

Total RNA from Guy11 and Δ*Mogln2* mutants was extracted using a Magen Kit as described previously [48]. The RNA integrity was assessed using the RNA Nano 6000 Assay Kit of the Bioanalyzer 2100 system (Agilent Technologies, California, CA, USA). cDNA libraries were constructed, and Illumina sequencing was performed on (Novaseq platform). Isolation of poly(A) mRNA from total RNA and construction of cDNA libraries were performed according to methods described previously [51]. After removing low-quality raw reads, the clean reads from each library were aligned to the transcript sequences of the *Magnaporthe oryzae* isolate 70-15 using bowtie2 (v2.3.4.1) [52], and the average mapping rate was 80.41%. The expression abundance was calculated by RSEM (v1.3.1) [53] with default parameters, and the TPM values of transcripts were exported to DESeq2 (v1.29.16) [54] for differential expression analysis. A gene was defined as being a differentially expressed gene (DEG) in the case of:

i. a minimum 2-fold difference in gene expression between the control Guy11 and the Δ*Mogln2* (|log_2_FC| > 1);
ii. a maximum false discovery rate (FDR) of 0.01 (FDR < 0.01).

### 2.7. High-Performance Liquid Chromatography (HPLC) Assays

Samples for glutamine for other amino acid tests were prepared by culturing the Guy11 and the Δ*Mogln1*, Δ*Mogln2*, and Δ*Mogln3* at 28 °C in liquid CM medium for three days and transferring to minimal medium for an additional two days with agitation (110 rpm). The strains were then filtered out, rinsed with sterilized double-distilled water, and frozen in liquid nitrogen. The dried hyphae tissues from the respective strains were ground into powders using a mortar and pestle. The grinded hyphae generated from the Guy11 and the *MoGLN* mutants were separately weighed into 2 mL Eppendorf tubes (EP-tubes) containing 50 μmol/L BTI acetonitrile. A total of 25 μL of pyridine aqueous solution (50 μmol/L) was added and mixed well. The samples were then incubated at 50 °C for 4 h. A total of 200 μL of 6 mol/HCl was then added, followed by hydrolyzation at 110 °C for 24 h. After the hydrolysis was completed, samples were dried with nitrogen and hydrolyzed with 100 μL of acetonitrile-pyridine-triethylamine-water (10:5:2:3) buffer. Then, 20 μL PITC (phenyl isothiocyanate) was added and incubated at 50 °C for 1 h. Following this, 250 μL of the samples were pipetted into a clean 2.0 mL EP tube, containing 750 μL 0.02 mol/L HCL and 200 μL N-hexane to remove impurities. The lower layer was then carefully transferred to the new 2.0 mL EP tubes for detection of glutamine. The quantifying services were performed and completed by Qingdao Sci-tech Innovation Quality Testing Co. Ltd., Qingdao, China.

For detection of other amino acids in Guy11 and Δ*Mogln2* mutants, ACQUITY UPLC^®^ BEH C18 column (2.1 × 100 mm, 1.7 μm, Waters, Milford, MA, USA) model HPLC instrument was used. Injection volume was 5 μL, and column temperature was 40 °C. Mobile phase A used 10% methanol water (containing 0.1% formic acid) and mobile phase B used 50% methanol water (containing 0.1% formic acid). The gradient elution conditions were 0~6.5 min, 10~30% mobile phase B; 6.5~7 min, 30~100% mobile phase B; 7~8 min, 100% mobile phase B; 8~8.5 min, 100~10% mobile phase B; 8.5~12.5 min, 10% mobile phase B. Flow rate was 0~8.5 min, 0.3 mL/min and 8.5~12.5 min, 0.3~0.4 mL/min. Mass spectrometry conditions were as follows: electrospray ionization (ESI) source, positive ionization mode. The ion source temperature was 500 °C, the ion source voltage was 5500 V, the collision gas was 6 psi, the curtain gas was 30 psi, and the atomization gas and auxiliary gas were both 50 psi. The quantifying services were performed and completed by Fuzhou Beiruisi Biotechnology Co. Ltd., Fuzhou, China.

### 2.8. Microscopy

To observe conidiophore development, conidia shapes, appressorium formation on inductive surfaces, appressorium penetration, and invasive hyphae development, an Olympus DP80 light microscope (Tokyo, Japan) was used, while GFP localization assays were examined using a confocal microscope equipped with Nikon A1 plus instrument (Nikon, Tokyo, Japan).

### 2.9. Bioinformatic Analysis

To identify MoGln1, MoGln2, and MoGln3 in the *M. oryzae,* the *S. cerevisiae* Gln1 amino acid sequence was used to perform a blastP search in the *M. oryzae* genome KEEG database (http://www.kegg.jp/kegg-bin/show_organism?org=mgr, accessed on 10 January 2020). The Gln1, Gln2, and Gln3 amino acid ortholog sequences from different fungi were obtained from (www.ncbi.nlm.nih.gov/blast, accessed on 10 January 2020) using the blast algorithm [55]. Domains were predicted by Pfam (http://pfam.janelia.org/, accessed on 10 January 2020) and presented using IBIS 1.0.3 software [56]. Sequence alignment was performed using MEGA v6, while phylogenetic tree was generated using the Maximum-Likelihood method, with branches of the tree tested with 1000 bootstrap replicates. The accession number for amino acid sequences used for phylogenetic analysis is as follows: MoGLN1 (XP_003709618); MoGLN2 (XP_003719336); MoGLN3 (XP_003721264); NcGLN1 (XP_960904); SsGLN1 (XP_001588876); FoGLN1 (XP_018240222); FgGLN1 (XP_011319217); TrGLN1 (XP_006967001); AfGLN1 (XP_023088587); AniGLN1 (XP_661763); UmGLN1 (XP_011390105); ScGLN1 (ONH79708); FfGLN1 (XP_023431521); AfGLN3 (RAQ56449); FgGLN3 (XP_011315791); TrGLN3 (XP_006967843); UmGLN3 (XP_011392295); AniGLN3 (XP_664258); NcGLN3 (XP_965073); SsGLN1 (XP_001593468).

## 3. Results

### 3.1. Identification of Glutamine Synthetase in M. oryzae

**Figure 1:**
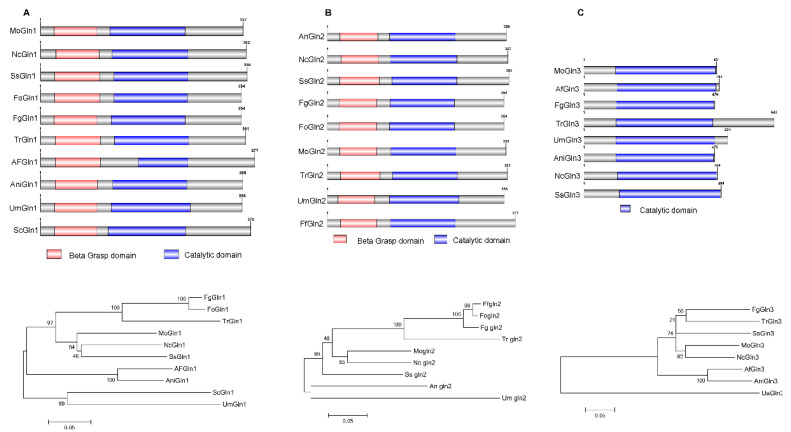
Domain architecture and phylogeny of *M. oryzae* glutamine synthetase in different fungi: (**A**) Gln1 domain architecture and maximum likelihood based phylogenetic outlook in different fungi; (**B**) Gln2 domain architecture and maximum likelihood phylogenetic analysis in fungi groups; (**C**) Domain architecture of Gln3 and the maximum likelihood phylogeny in different fungi. Maximum likelihood phylogeny for the glutamine synthetase amino acids of different fungi was tested with 1000 bootstrap replicates.

To obtain sequences for *M. oryzae* glutamine synthetase genes, referred to here as *MoGLN*, the amino acid sequence of glutamine synthetase gene (*GLN1*) from *Saccharamycess cerevisiae* was used to conduct a blastP search in Kyoto Encyclopedia of Genes and Genome (KEGG) resource section for *M. oryzae* (http://www.kegg.jp/kegg-bin/show_organism?org=mgr, accessed on 10 January 2020). Three putative amino acid sequences that encode glutamine synthetase were identified and were named, based on a previous study, as MoGln1 (MGG_06888), MoGln2 (MGG_14279), and MoGln3 (MGG_02538) [57]. The three obtained MoGln amino acids were used for a blastP search to identify glutamine synthetase amino acid sequences in other fungi in the Fungi and Oomycetes genomics resources database (http://fungidb.org/fungidb/, accessed on 10 January 2020) and National Centre of Biotechnology Information (https://www.ncbi.nlm.nih.gov/, accessed on 10 January 2020). The retrieved amino acid sequences were then used to conduct Pfam-based domain prediction. Results obtained showed that Gln1 and Gln2 contained two conserved domains—glutamine synthetase, a catalytic domain, and glutamine synthentase, a beta-Grasp domain—while Gln3 contained a single glutamine synthentase—beta-Grasp domain—and this domain was conserved in fungi (Figure 1A–C). Phylogenetic analysis revealed that MoG1n1, MoG1n2, and MoG1n3 shared a close ancestor with Gln1, G1n2, and G1n3 of *Neurospora crassa* (Nc), respectively (Figure 1A–C).

### 3.2. Expression of MoGLN Genes at Different Developmental Stages of M. oryzae

**Figure 2:**
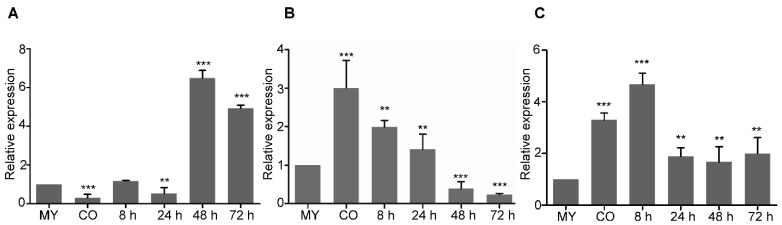
Phase-specific expression of the three *MoGLN* genes at various development stages of *M. oryzae*. (**A**) phase-specific expression of *MoGLN1;* (**B**) phase-specific expression of *MoGLN2;* (**C**) phase-specific expression of *MoGLN3*. The phase-specific expression of the three *MoGLN* genes was quantified by quantitative real-time (QRT)-PCR after synthesis of cDNA in each developmental stage. The *ACTIN* gene (MGG_03982) was used for internal control for normalization, and the expression level of each gene at the mycelial stage was considered 1 for further comparisons. The qPCR results were obtained from three independent biological replications with three technical replicates. Error bars represent standard deviations. Asterisks indicate statistically significant differences (**, *p* < 0.01; ***, *p* < 0.001; one-way ANOVA was used to analyze data with Tukey’s multiple-comparison test in GraphPad Prism 8).

It was initially assumed that through-checking the expression pattern of the three *MoGLN* genes at various developmental stages of the fungus would provide information on their likely roles. Using the WT strain, we quantified the expression level of these genes at conidia and in the *planta* stage (8 h, 24 h, 48 h, and 72 h). The Guy11 mycelia stage was used as a control, and in the *planta* stages, 21-day-old rice leaves were sprayed with Guy11 spores. The expression of *MoGLN1* was found to be higher at the late infection stages of fungus, with the fold increases of −0.7, 0.1, −0.5, 5.5, and 4.0 at sporulation, 8 h, 24 h, 48 h, and 72 h, respectively (Figure 2A). For *MoGLN2,* we noted an elevated expression at the sporulation stage, with fold increases of 2.0, 1.0, 0.4, −0.6, and −0.8 at conidiation, 8 h, 24 h, 48 h, and 72 h, respectively (Figure 2B). Finally, our stage-specific qPCR analysis established that the transcripts levels of *MoGLN3* were high at the early infection stage, with fold increases of 2.4, 3.8, 0.8, 0.6, and 1.0 at the asexual stage, 8 h, 24 h, 48 h, and 72 h, respectively (Figure 2C). To validate the exact functions of the three *MoGLN* genes, their respective deletion mutants were generated, and phenotype was characterized.

### 3.3. Generation of ΔMogln1, ΔMogln2, and ΔMogln3 Deletion Strains

**Figure 3:**
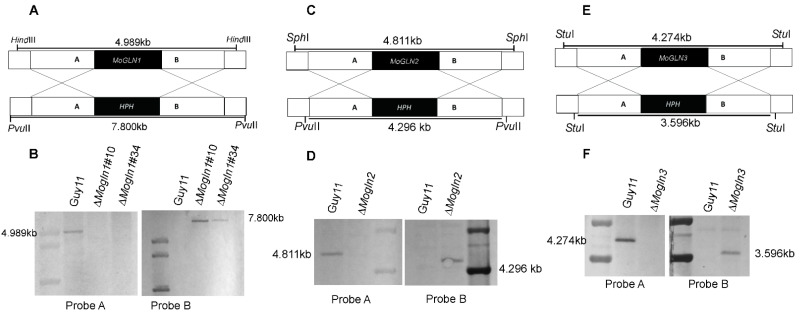
Southern blot analysis to confirm *MoGLN* deletion mutants. (**A**,**B**) Sketch representation of deletion of *MoGLN1* in the *M. oryzae* genome and southern blot analysis of the gene knockout mutants and WT Guy11 via *MoGLN1* ORF probe A and hygromycin phosphotransferase (*HPH*) probe B. (**C**,**D**) Sketch representation of deletion of *MoGLN2* in *M. oryzae* genome and southern blot analysis of the gene knockout mutant and WT Guy11 via *MoGLN2* ORF probe A and hygromycin phosphotransferase (*HPH*) probe B. (**E**,**F**) Sketch representation of deletion of *MoGLN3* in the *M. oryzae* genome and southern blot analysis of the gene knockout mutant and the WT Guy11 using *MoGLN3* ORF probe A and hygromycin phosphotransferase (*HPH*) probe B.

To establish the exact roles of the *MoGLN* genes in development and pathogenicity of the rice blast fungus, we generated their respective deletion mutants using a homologous recombination approach by replacing each of *MoGLN1, MoGLN2,* and *MoGLN3* open reading frame (ORF) with the hygromycin phosphotransferase (*HPH*) gene. To generate the gene deletion constructs, the upstream (A fragment) and downstream (B fragment) flanking regions of *MoGLN1, MoGLN2,* and *MoGLN3* were amplified and ligated with 5′ and 3′ split parts of a hygromycin-resistant gene. Each of the constructs was separately transformed in Guy11 protoplast and screened on TB3 medium containing hygromycin resistance. Putative transformants for *MoGLN1, MoGLN2,* and *MoGLN3* deletions were screened by PCR with gene-specific ORF primer pairs (Table S1), and successful deletion of *MoGLN1, MoGLN2* and *MoGLN3* was subsequently confirmed using southern blot assays. Results obtained after confirmation assays showed *MoGLN1, MoGLN2,* and *MoGLN3* open reading frame (ORF) were successfully replaced with a single integration of hygromycin phosphotransferase (*HPH*) to generate Δ*Mogln1* (Figure 3A,B)*,* Δ*Mogln2* (Figure 3C,D), and Δ*Mogln3* (Figure 3E,F).

### 3.4. MoGLN2 Contributes to Vegetative Growth in M. oryzae

**Figure 4:**
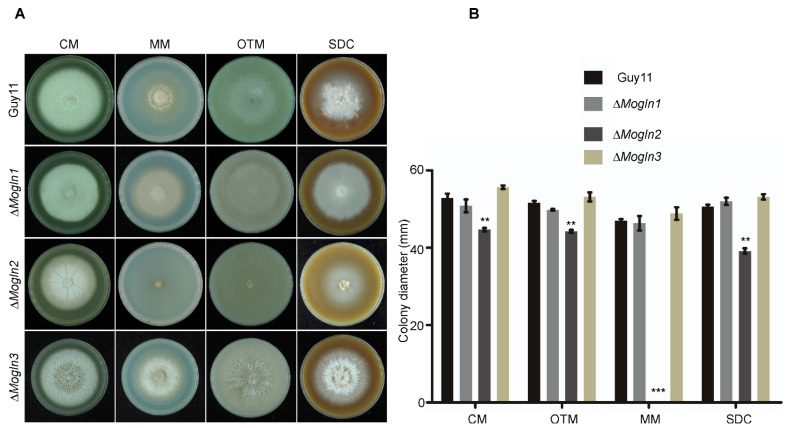
*MoGLN2* is required for vegetative growth in *M. oryzae*. (**A**) Photographs showing radial and aerial hyphal growth of the wild-type (WT) and the three mutants. Mycelial plugs inoculated on CM, MM, OTM, and SDC were cultured in the dark at 28 °C, and photograph taken after eight days. (**B**) Bar graphs showing the difference in radial growth between the WT and the three *MoGLN* mutants. The error bar represents the standard deviation of three independent replicates, while the double asterisk shows significant difference (**, *p* < 0.01; ***, *p* < 0.001; one-way ANOVA was used to analyze data with Tukey’s multiple-comparison test in GraphPad Prism 8).

To investigate the contribution of *MoGLN* genes in vegetative growth of *M. oryzae*, we cultured the three respective *MoGLN* mutants on CM (complete medium), MM (minimal medium), OTM (oatmeal medium), and SDC (straw decoction and corn medium) and observed their growth. After eight days of inoculation, we established no significant difference in mycelial growth in terms of colony diameter between the wild-type Guy11, Δ*Mogln1,* and Δ*Mogln3* on four different types of medium (Figure 4A,B). However*,* the growth of Δ*Mogln2* mutants was remarkably reduced in CM, OTM, and SDC, and the mutant failed to grow on MM medium (Figure 4A,B). In addition, the Δ*Mogln2* exhibited poor development of aerial hyphal on SDC and OTM when compared to the wild-type Guy11, Δ*Mogln1,* and Δ*Mogln3* strains (Figure 4A,B). Introduction of *MoGLN2* gene into the Δ*Mogln2* mutant restored the growth defects of Δ*Mogln2* on CM, MM, OTM, and SDC. These results indicated that *MoGLN2* is required for proper vegetative growth in *M. oryzae*.

### 3.5. Glutamine Auxotroph in Rice Blast Fungus Occurs via Inactivation of MoGLN2

**Figure 5:**
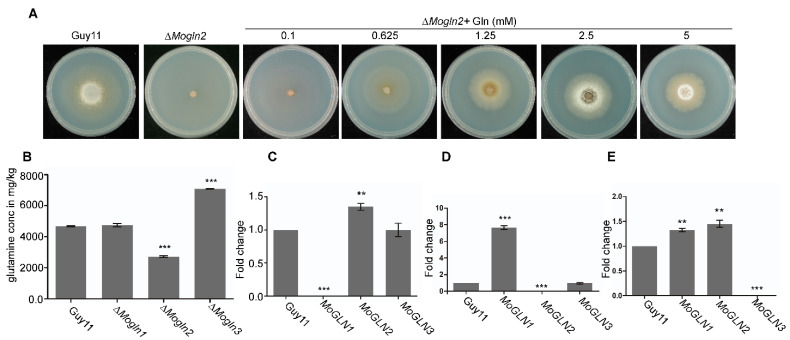
Exogenous glutamine restores growth defects of Δ*Mogln2* on MM medium. (**A**) Radial growth of Δ*Mogln2* mutant on MM medium supplemented with different concentrations of glutamine. The experiment was repeated three times with similar results obtained. (**B**) Statistical representation of intracellular glutamine levels detected in WT and the three *MoGLN* mutants. Error bars represent standard deviations obtained from two independent tests. Data were analyzed using GraphPad Prism 8; asterisks indicate statistically significant differences (**, *p* < 0.01; ***, *p* < 0.001; based on one-way ANOVA with Tukey’s multiple-comparison test). (**C**) Graph showing the expression of *MoGLN2* and *MoGLN3* in Δ*Mogln1* mutant; (**D**) graphical representation of expression pattern of *MoGLN1* and *MoGLN3* in the Δ*Mogln2* mutant; (**E**) expression pattern of *MoGLN1* and *MoGLN2 in* Δ*Mogln3* mutant. The actin gene was used as a control. Data for statistical analysis were obtained after performing three independent biological replicates. Error bars represent standard deviations. Asterisks indicate statistically significant differences (**, *p* < 0.01; ***, *p* < 0.001; one-way ANOVA was used to analyze data with Tukey’s multiple-comparison test in GraphPadPrism 8).

In *Aspergillus nidulans*, deletion of the glutamine synthetase gene resulted in the mutant cells requiring glutamine for growth in MM medium [34]. Because the Δ*Mogln2* mutant was attenuated in MM, we speculated the mutant cells lacked sufficient glutamine levels required for growth. To test this idea, we first tested the growth of Δ*Mogln2* in MM medium supplemented with different concentrations of glutamine (0.1 mM, 0.625 mM, 1.25 mM, 2.5 mM, and 5 mM). Our results showed that exogenous glutamine could restore growth of Δ*Mogln2* on MM medium, with more aerial hyphal being observed at high concentrations of glutamine (Figure 5A). Since the growth defect of Δ*Mogln2* on MM medium was attributed to insufficient glutamine levels, we detected intracellular glutamine in the mycelia of the three *MoGLN* mutants. No significant change in glutamine levels was recorded in Δ*Mogln1,* while glutamine levels were significantly lower and higher in Δ*Mogln2* and Δ*Mogln3* strains, respectively (Figure 5B). We then sorted to determine if deletion of one *MoGLN* gene affected the expression of the remaining two genes. We observed an up-regulation of *MoGLN2* in Δ*Mogln1* mutant, with expression of *MoGLN3* being unaffected (Figure 5C). The expression level of *MoGLN1* was higher in the Δ*Mogln2* mutant, with no detectable change in expression of *MoGLN3* (Figure 5D). Lastly, both *MoGLN1* and *MoGLN2* were up-regulated in Δ*Mogln3* mutant (Figure 5E). These expression patterns showed that glutamine levels in Δ*Mogln1* were from *MoGLN2,* low levels of glutamine in the Δ*Mogln2* mutant were from *MoGLN1,* and the highest glutamine levels in Δ*Mogln3* were from *MoGLN1* and *MoGLN2.* These results suggest that both *MoGLN1* and *MoGLN2* could be involved in de novo glutamine biosynthesis. However, the level of glutamine produced by *MoGLN1* is not sufficient enough to sustain normal cellular function. Further evidence for the involvement of *MoGLN1* in glutamine biosynthesis was reported in Δ*Moasd4* after glutamine levels were significantly lowered in Δ*Moasd4* upon deletion of *MoGLN1* in Δ*Moasd4* [57]. Overall, we conclude that glutamine auxotroph in rice blast fungus only occurs via deletion of *MoGLN2.*

### 3.6. MoGLN2 Is Required for Asexual Reproduction in M. oryzae

**Figure 6:**
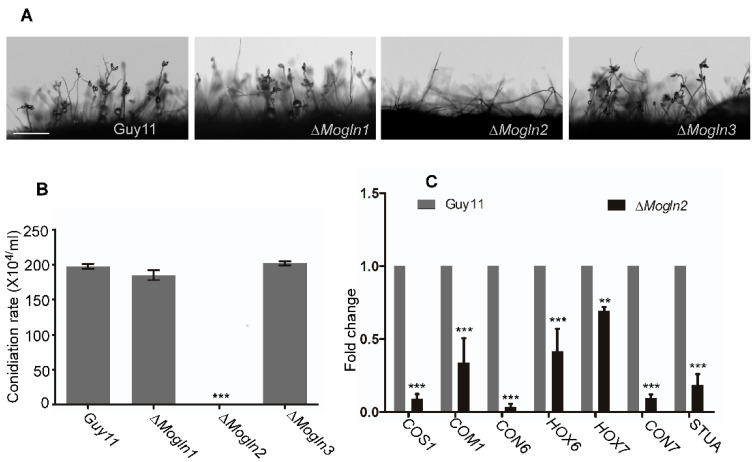
MoGln2 plays an important role in asexual reproduction in *M. oryzae*. (**A**) Represents conidiophore development and spore formation capacity of strains cultured on rice bran medium for 10 days. Bar, 10 μm. (**B**) Graph showing quantification of spores from Guy11, Δ*Mogln1*, Δ*Mogln2,* Δ*Mogln3* strains on rice bran medium. The Δ*Mogln2* mutant failed to produce spores. (**C**) Quantitative RT-PCR analysis showing the expression of conidiation-related genes in the WT and Δ*Mogln2* mutants. The expression was normalized actin gene (MGG_03982). Results are means obtained from three independent replicates. Error bars represents standard deviations. Asterisks indicate statistically significant differences (**, *p* < 0.01; ***, *p* < 0.001; one-way ANOVA was applied to analyze data with Tukey’s multiple-comparison test in GraphPad Prism 8).

To determine the roles of the three *MoGLN2* genes in sexual reproduction in rice blast fungus, the wild-type Guy11 and the three mutant strains were cultured on sporulation rice bran medium for 10 days, and then conidiophore development and conidia formation evaluated. The wild-type strain, Δ*Mogln1,* and Δ*Mogln3* produced similar conidiophores and an equal number of spores (Figure 6A,B). No conidia or conidiophores was formed in the cultures of Δ*Mogln2* mutants (Figure 6A,B). Since rice bran medium could not initiate conidiation in Δ*Mogln2*, we tried different conidiation media, including OTM and SDC. Neither of these activated conidiation in the Δ*Mogln2* mutant. As glutamine synthetase catalyzes the biosynthesis of glutamine, we supplemented rice bran, OTM, and SDC media with different concentrations of glutamine (1 mM, 2 mM, 5 mM, 10 mM, 20 mM, 40 mM, and 60 mM). None of these glutamine concentrations rescued the conidiation defects in the Δ*Mogln2* mutant. We then performed quantitative real-time PCR (qRT-PCR) analysis to check the transcript levels of conidiation-related genes, including *COS1, COM1, CON6, CON7, HOX6, HOX7,* and *STUA*. The expression of these genes was found to be significantly reduced in the Δ*Mogln2* mutant (Figure 6C), indicating that *MoGLN2* regulates conidiogenesis in rice blast fungus through controlling the expression of conidiation-related genes. In summary, these results shows that *MoGLN2* plays an important role in asexual reproduction in rice blast fungus.

### 3.7. MoGLN2 Is Important for Appressorium Formation in M. oryzae

**Figure 7:**
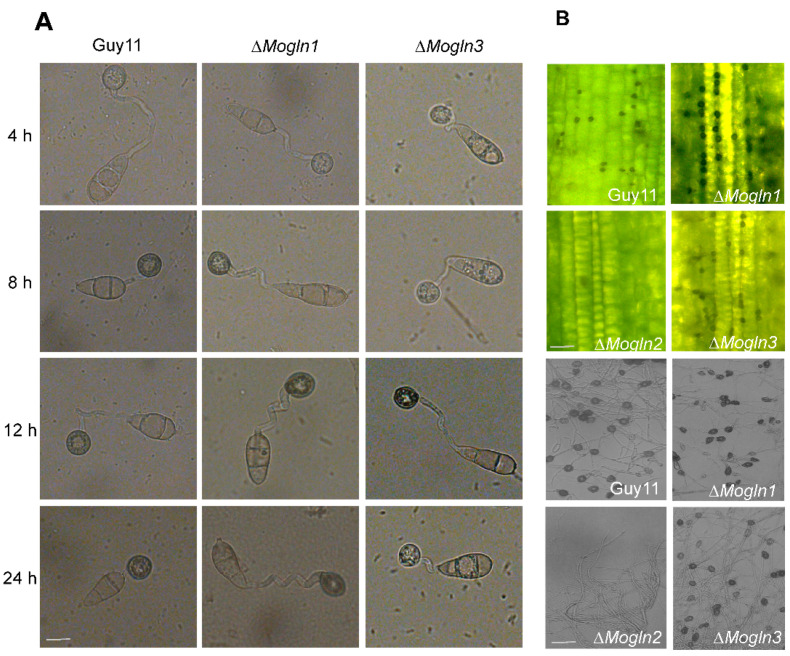
MoGln2 plays a crucial role in appressorium formation in rice blast fungus. (**A**) Bright field micrographs of the appressoria formed by WT, Δ*Mogln1*, and Δ*Mogln3* mutants on inductive hydrophobic cover slips. Conidia from WT, Δ*Mogln1*, and Δ*Mogln3* mutants were inoculated on a hydrophobic cover slip, and appressoria formation was observed at 4 h, 8 h, 12 h, and 24 h time intervals. Scale bar = 10 μm. (**B**) An appressorium-like structure formed on a hydrophobic surface and barley leaves. Mycelia plugs derived from WT, Δ*Mogln1*, Δ*Mogln2,* and Δ*Mogln3* were inoculated on 10-day-old barley leaves, and inductive hydrophobic cover slips; appressorium-like structure formation was observed after 30 h. Scale bar = 10 μm. The Δ*Mogln2* mutant failed to form appressorium-like structures both on barley leaves and hydrophobic cover slips.

In rice blast fungus, infection occurs when *M. oryzae* spores land on the rice leaf surface and germinate into a specialized structure called appressorium. Besides rice leaves, appressorium also forms when *M. oryzae* spores encounter a hydrophobic surface, as it mimics the rice leaf surface. Because Δ*Mogln1* and Δ*Mogln3* produced spores, their respective spores alongside with those from the Guy11 wild strain were inoculated on an artificial hydrophobic surface, and spore germination and appressorium formation were examined at 4 h, 8 h, 12 h, and 24 h time intervals. Both Δ*Mogln1* and Δ*Mogln3* produced normal appressoria that was indistinguishable from the wild-type strain (Figure 7A), thus confirming that *MoGLN1* and *MoGLN3* are not required for appressorium formation in *M. oryzae*. It has been reported that rice blast fungus can form appressoria from its hyphae [58]. We then evaluated appressorium formation using mycelia plugs of Guy11, Δ*Mogln1,* Δ*Mogln2,* and Δ*Mogln3* strains by first observing appressorium-like structure formation on an artificial hydrophobic surface. After 24 h, no appressorium-like structure formed on a hydrophobic surface inoculated with mycelia from Δ*Mogln2*; in contrast, the Δ*Mogln1* and Δ*Mogln3* formed appressorium-like structures similar to the Guy11 strain (Figure 7B). Furthermore, we examined appressorium-like structures on barley leaves inoculated with the three *MoGLN* mutants; similarly, Δ*Mogln2* failed to form appressorium-like structures on barley leaves, but Δ*Mogln1* and Δ*Mogln3* and the Guy11 strain formed appressorium-like structures (Figure 7B). Collectively, we conclude that *MoGLN2* is important for appressorium formation in rice blast fungus.

### 3.8. MoGLN2 Is Essential for Full Virulence in M. oryzae

**Figure 8:**
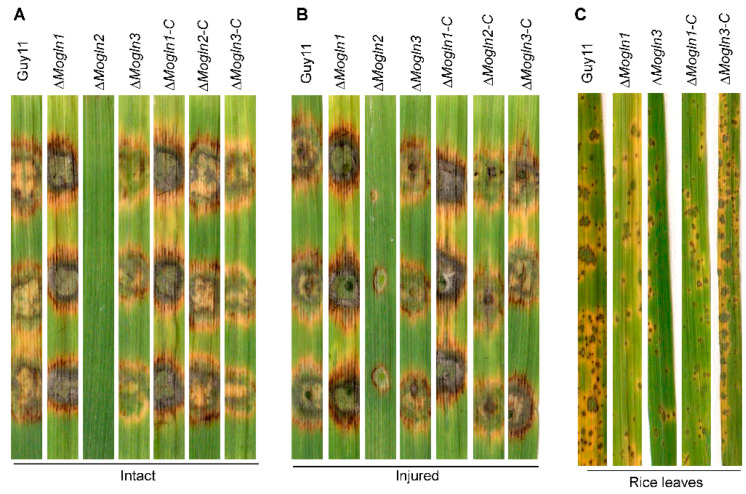
*MoGLN2* plays an important role in promoting the infections of *M. oryzae*. (**A**,**B**) Δ*Mogln2* failed to induce hyphae-mediated blast lesions on intact and injured barley leaves. (**C**) Rice leaves bearing blast lesions of Δ*Mogln1* and Δ*Mogln3* mutant spores. Both barley and rice leaf images were taken seven days post inoculation.

To establish the role played by different subunits of glutamine synthase in the pathogenicity of rice blast fungus, we first examined the virulence of Δ*Mogln1*, Δ*Mogln2,* and Δ*Mogln3* by inoculating their mycelial plugs on 10-day-old barley leaves. Seven days after inoculation, Δ*Mogln2* failed to cause disease symptoms both on intact and injured barley leaves, while the wild-type, Δ*Mogln1*, and Δ*Mogln3* produced large similar blast lesions on barley leaves (Figure 8A,B). Since Δ*Mogln1* and Δ*Mogln3* strains could produce spores, we harvested the Guy11, Δ*Mogln1,* and Δ*Mogln3* spores from 10-day-old rice bran cultures used to spray 3-week-old seedlings of the susceptible rice variety CO39. Our results showed that both Δ*Mogln1* and Δ*Mogln3* strains produced necrotic blast lesions on rice leaves similar to the wild-type strains (Figure 8C). Based on these results, we conclude that amongst the three *MoGLN* genes, *MoGLN2* is solely involved in the pathogenicity of rice blast fungus.

### 3.9. MoGLN1 and MoGLN3 Are Not Involved in Appressorium Penetration and Infectious Hyphal Growth

**Figure 9:**
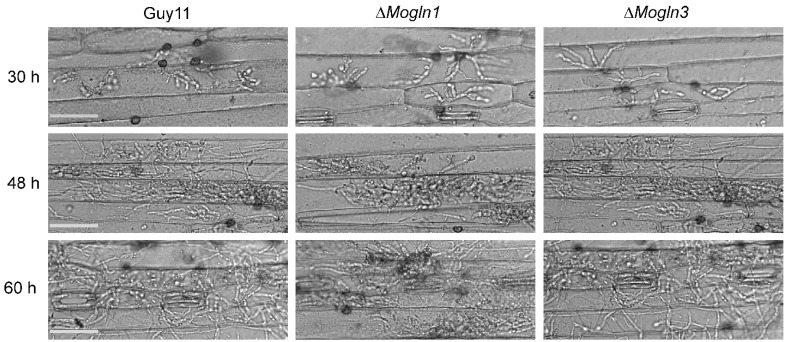
Bright field micrographs showing the development of invasive hyphal growth of WT, Δ*Mogln1*, and Δ*Mogln3*. Spore suspensions from Guy11, Δ*Mogln1*, and Δ*Mogln3* strains were inoculated on 10-day-old barley leaves, and invasive hyphal growth was observed at 30 h, 48 h, and 72 h. Bar= 20 µm.

Appressorium penetration is an essential process that allows the fungus to get inside the host cell and cause infection. Appressorium-mediated penetration occurs when turgor pressure builds up within appressorium, which is used to breach the host surface [59]. Owing to the fact that Δ*Mogln1* and Δ*Mogln3* spores could form appressorium on the hydrophobic coverslip, we monitored appressorium penetration and subsequent invasive hyphal formation by inoculating conidia from Guy11, Δ*Mogln1,* and Δ*Mogln3* on 10-day-old barley. After 30 h of inoculation, the majority of the appressoria of Guy11, Δ*Mogln1,* and Δ*Mogln3* strains had penetrated the barley cells and started forming invasive hyphae (Figure 9). At 48 h, the invasive hyphal of the three strains had spread and colonized the adjacent cells (Figure 9), clearly showing that deletion of either *MoGLN1* or *MoGLN3* did not affect these processes. These results indicate that both *MoGLN1* and *MoGLN3* are not required for appressorium penetration and invasive hyphal formation in rice blast fungus.

### 3.10. Cell Wall Integrity Is Impaired in the ΔMogln2 Deletion Mutant

**Figure 10:**
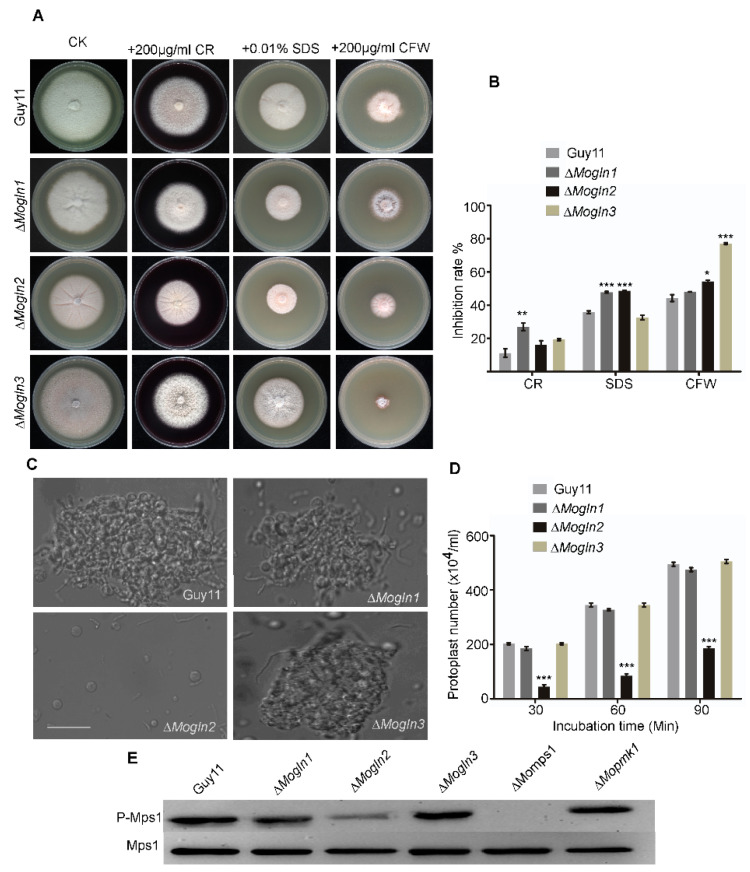
*MoGLN2* is essential for maintenance of cell wall integrity in *M. oryzae*. (**A**) The Guy11 and *MoGLN* mutants were cultured on CM medium supplemented with (200 µg/mL CR, 0.01% SDS, and 200 µg/mL CFW) at 28 °C for 8 days before being photographed. (**B**) Graph showing inhibition rate of WT and mutant strains. Inhibition rate was compared to the growth rate of each untreated control (Inhibition rate = (the diameter of untreated strain − the diameter of treated strain)/(the diameter of untreated strain × 100%)). Three independent repeats were performed, with similar results obtained. (**C**) Light microscopic examination of protoplast release after treatment with cell-wall-degrading enzymes for 30 min, 60 min, and 90 min at 28 °C. Bar= 10 µm (**D**) Graphical representation of protoplast release assay for the WT and three *MoGLN* mutants. (**E**) Phosphorylation of *MoMps1* in WT and three *MoGLN* mutants, Δ*Mops1,* and Δ*Mopmk1*. Proteins were prepared from mycelia inoculated in liquid CM, and the phosphorylated *MoMps1* was detected by binding of the antiphospho-p44/42 antibody, with the Mpk1 antibody as a control. The phosphorylation level of MoMps1 in the Δ*Mogln2* strains indicated the reduced activation of *MoMps1*. Statistical results for growth inhibition rate and protoplast results were obtained from at least three independent replicates. Error bars represent standard deviations. Asterisks indicate statistically significant differences (*, *p* < 0.005 **, *p* < 0.01, ***, *p* < 0.001; one-way ANOVA was used to analyze data with Tukey’s multiple-comparison test in GraphPad Prism 8).

To investigate the contribution of three *MoGLN* genes in fostering cell wall integrity in rice blast fungus, we first monitored and measured the vegetative growth of three *MoGLN* mutants on CM medium amended with cell wall stressors Calcofluor white (CFW) [60,61], Sodium Dodecyl Sulfate (SDS) [62,63], and Congo Red (CR) [64]. After eight days post inoculation, our results showed only Δ*Mogln1* mutant was highly inhibited on plates containing CR. On SDS medium, both Δ*Mogln1* and Δ*Mogln2* were highly inhibited, while on CM medium containing CFW, Δ*Mogln2* was slightly inhibited, with Δ*Mogln3* being highly sensitive (Figure 10A,B). We performed additional tests to conclusively determine which among the genes is involved in the maintenance of cell wall integrity. We examined the effects of lytic enzymes (10 mg/mL lysing enzymes) on the three Δ*Mogln* mutants. Fewer protoplasts were generated by the Δ*Mogln2* mutant compared to wild-type Guy11, Δ*Mogln1*, and Δ*Mogln3* strains (Figure 10C,D), indicating either the cell wall structure was altered, making it less resistant to degradation by lytic enzymes, or the membrane and cell wall were breached as a result of excess rupture, thus leading to poor protoplast recovery. In rice blast fungus, reduced Mps1 phosphorylation was previously reported in a mutant with altered cell wall integrity [65]. Therefore, we performed western blot assay to determine the phosphorylation of Mps1 in the three *MoGLN* mutants. Mps1 phosphorylation remained unchanged in the Δ*Mogln1* and Δ*Mogln3* strains, and it was remarkably reduced in the Δ*Mogln2* mutant, indicating cell wall defects associated with loss of *MoGLN2* (Figure 10E). Taken together, these results indicate *MoGLN2* is important in maintaining cell wall integrity in rice blast fungus.

### 3.11. Intracellular Levels of Other Amino Acids Were Higher in ΔMogln2 Mutant

In *Saccharomyces cerevisiae,* reduced glutamine levels detected in a hypo-osmorality *GLN1* mutant resulted in increased intracellular amounts of the other amino acids, except for proline [66]. To confirm whether low levels of glutamine affected the concentration of other amino acids in the Δ*Mogln2* mutant, the Guy11 and Δ*Mogln2* strains were cultured in liquid CM medium for three days and then transferred to MM medium for an additional three days. Mycelial samples were then used to detect the concentration of other amino acids. The steady-state intracellular pools of almost all the amino acids detected were found to have increased in Δ*Mogln2* (Table 1). Analysis of RNA sequencing data for the Guy11 and Δ*Mogln2* mutants showed that increased intracellular amino acid levels in Δ*Mogln2* correlated with the expression of genes related to amino acid biosynthesis, as RNA sequencing transcriptome data showed that the majority of genes, including those involved in translation, amino acid activation, tRNA aminoacylation, tRNA aminoacylation for protein translation, amide biosynthetic process, and peptide biosynthetic process, were found to be differentially up-regulated in the Δ*Mogln2* mutant (Figure 13A). This confirms that reduced glutamine levels in Δ*Mogln2* resulted in increased biosynthesis of other amino acids.

### 3.12. ΔMogln2 Is Hypersensitive to Oxidative Stress

**Figure 11:**
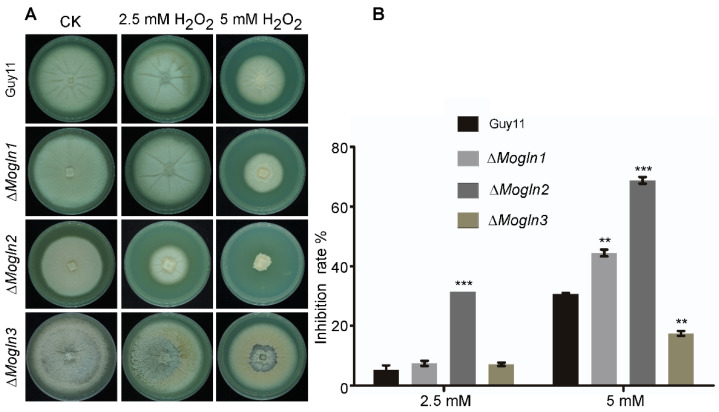
Sensitivity of the three *MoGLN* mutants to H_2_O_2_. (**A**) Growth phenotype of the WT and *MoGLN* mutants under oxidative stress. The WT and three mutant strains were inoculated on CM agar medium with or without 2.5 mM H_2_O_2_ and 5 mM H_2_O_2_ and cultured at 28 °C for 10 days. (**B**) The colony diameters of the strain tested were measured, and statistical analysis was performed. The growth inhibition rate was compared to the growth rate of each untreated control (Inhibition rate = (the diameter of untreated strain − the diameter of treated strain)/(the diameter of untreated strain × 100%)). Three independent repeats were performed, with similar results obtained. Error bars denote the standard deviations from means obtained from three independent replicates. Asterisks indicate statistically significant differences (**, *p* < 0.01; ***, *p* < 0.001; one-way ANOVA was used to analyze data with Turkey’s multiple-comparison test in Graph Pad Prism 8).

In rice fungal pathogens, sensitivity to oxidative stress using hydrogen peroxide is well documented [67,68,69]. To investigate the contributions of the three *MoGLN* genes in oxidative stress tolerance in rice blast fungus, we observed the mycelia growth of the three respective mutants on CM medium amended with 2.5 mM and 5 mM concentrations of hydrogen peroxide (H_2_O_2_). We established that mycelial growth of the Δ*Mogln1* mutant was moderately inhibited on CM medium containing 5 mM H_2_O_2_, while the Δ*Mogln3* mutant exhibited less sensitivity both on 2.5 mM and 5 mM concentrations of H_2_O_2_ relative to the WT strain (Figure 11A,B). However, the Δ*Mogln2* mutant was hypersensitive to both 2.5 mM and 5 mM H_2_O_2_ concentrations (Figure 11A,B), suggesting therefore that *MoGLN2* could be involved in the regulation of oxidative stress tolerance in rice blast fungus.

### 3.13. MoGLN2 Is Important for Melanin Biosynthesis in Rice Blast Fungus

**Figure 12:**
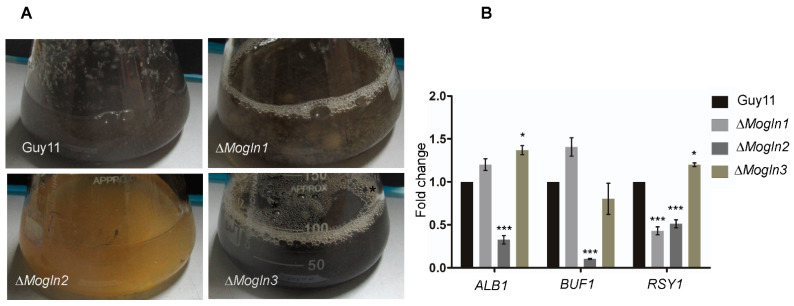
*MoGLN2* is required for hyphal melanization in *M. oryzae.* (**A**) Mycelial growth in liquid CM medium showing impaired hyphal melanization as a result of *MoGLN2* gene. (**B**) qRT-PCR analysis of the expression levels of genes important for melanin biosynthesis in mycelium grown in liquid CM. Error bars denote the standard deviations from means obtained from three independent replicates. Asterisks indicate statistically significant differences (*, *p* < 0.005; ***, *p* < 0.001; one-way ANOVA was used to analyze data with Tukey’s multiple-comparison test in GraphPad Prism 8).

In fungal pathogens, mycelia and appressoria undergo melanization; appressorial melanization is important for the normal functioning of the appressorium. After being cultured in liquid CM medium for five days, we observed a darkening of mycelial color for Guy11, Δ*Mogln1*, and Δ*Mogln3* strains, implying that Guy11, Δ*Mogln1,* and Δ*Mogln3* could be undergoing hyphal melanization. In contrast, no clearly visible darkening of mycelia was observed in culture inoculated with Δ*Mogln2* strains (Figure 12A). This prompted us to speculate that the failure of Δ*Mogln2* mycelia to form black pigmentation could be a result of the repression of genes important for melanization. We then performed qPCR analysis to confirm the expression of these genes in the three *MoGLN* mutants. As expected, *BUF1*, *RSY1,* and *ALB1* were found to be down-regulated in the Δ*Mogln2* mutant (Figure 12B), indicating that the melanization defect exhibited by the Δ*Mogln2* strain is a result of reduced expression of these genes. Based on these results, we conclude that *MoGLN2* plays a crucial role in the regulation of hyphal melanization in rice blast fungus.

### 3.14. Differentially Expressed in ΔMogln2 Mutant

**Figure 13:**
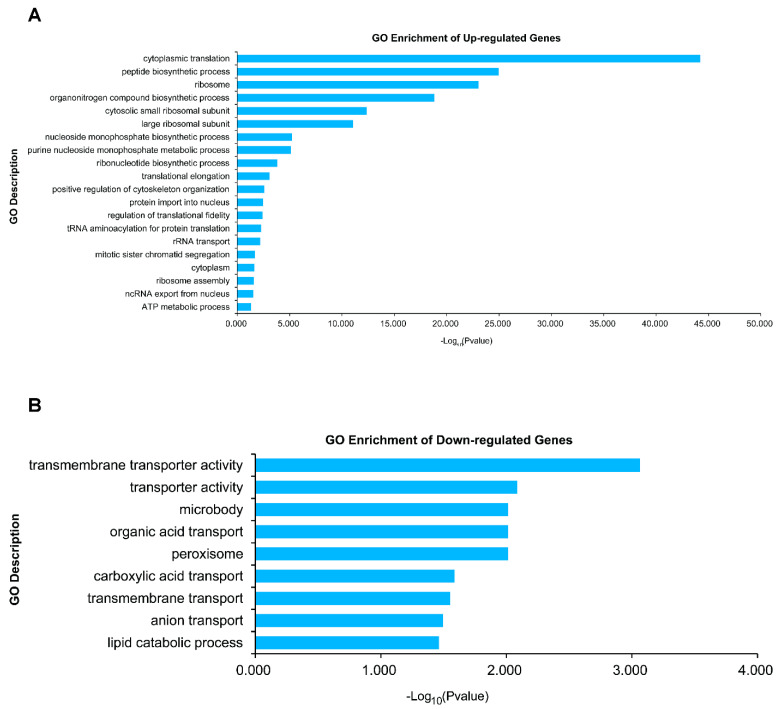
Molecular functions of the genes up-regulated (**A**) and down regulated (**B**) in Δ*Mogln2* at a two-fold expression threshold based on the Gene Ontology (GO) terms.

Considering the dramatic phenotype exhibited by Δ*Mogln2*, we performed RNA sequencing and analyzed the transcriptome data for Guy11 and Δ*Mogln2.* This was aimed at establishing which genes were differentially expressed after deletion of *MoGLN2.* We established that 3703 genes were differentially expressed, including 1819 and 1884 up-regulated and down-regulated genes, respectively (Figure S1A,B). Gene Ontology (GO) and KEGG enrichment analysis for the genes up-regulated and down-regulated in Δ*Mogln2* showed many enriched GO terms for up-regulated genes, including those that are involved as ribosome, translation, peptide biosynthetic process, purine nucleoside monophosphate metabolic process, ribonucleotide biosynthetic process, and obsolete cytosolic part (Figure 13A). KEGG analysis showed three enriched pathways, including Ribosome, oxidative phosphorylation’ and aminoacyl-tRNA biosynthesis (Figure S2A). For down-regulated genes, we obtained fewer enriched results. In GO enrichment analysis, we discovered the term transmembrane transporter activity, peroxisome, and lipid catabolic process were enriched (Figure 13B). Moreover, we only discovered two enriched pathways, including microbial metabolism in diverse environments and ABC transporters (Figure S2B).

### 3.15. Subcelullar Localization of MoGLN Genes

**Figure 14:**
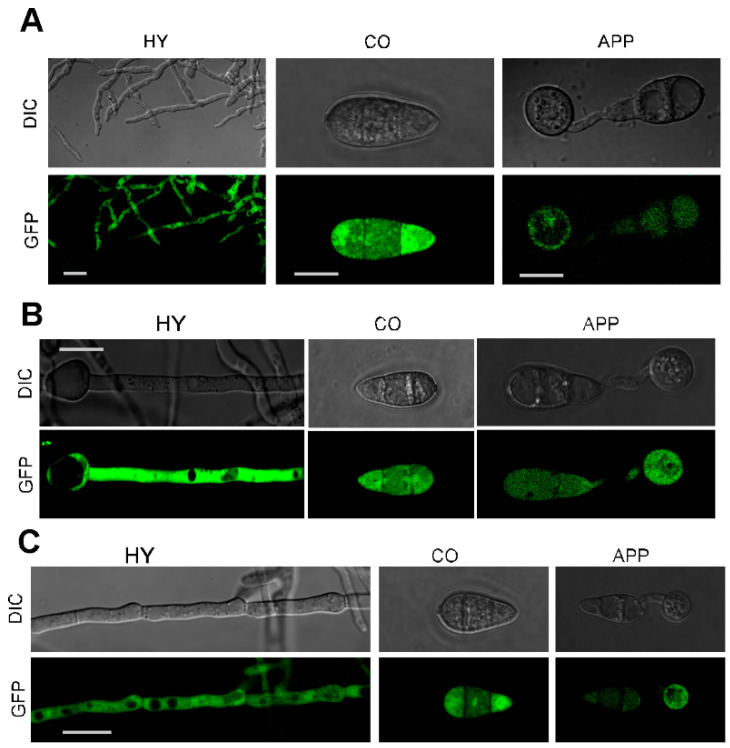
Subcellular localization of MoGln1, MoGln2, and MoGln3 in rice blast fungus. (**A**–**C**) The localization pattern of MoGln1-GFP, MoGln2-GFP, and MoGln3-GFP in hyphae, conidia, and appressorium. Localization of MoGln1-GFP, MoGln2-GFP, and MoGln3-GFP were examined by Nikon laser confocal, scale bar = 10 μm, MoGln1-GFP hyphae scale bar = 5 μm.

To evaluate the subcellular localization of MoGln1, MoGln2, and MoGln3 in rice blast fungus, the *MoGLN1*, *MoGLN2,* and *MoGLN3* genes, with their corresponding native promoters, were fused in the C-terminus region of GFP and cloned in the pKNTG vector containing neomycin-resistant genes [70]. The constructs were separately transformed in their respective mutant protoplast. Results obtained showed that MoGln1-GFP, MoGln2-GFP, and MoGln3-GFP were all targeted to the cytoplasm in growing hyphae, conidia, and appressorium (Figure 14A–C), thus indicating the three *MoGLN* genes are all located in the cytoplasm in rice blast fungus.

## 4. Discussion

Glutamine synthetase (GS) catalyzes the biosynthetic pathway involved in the synthesis of glutamine, and thus plays an important role in the assimilation of nitrogen. These enzymes have been extensively characterized in bacteria [41,71] and some filamentous fungi [34,72]. However, their functions in rice blast fungus remain uncharacterized. In this study, we identified and performed functional analysis of the three genes that encode glutamine synthetase (*GLN1, GLN2,* and *GLN3)* in *M. oryzae*. Upon deletion of each of the GS genes, we established that both *MoGLN1* and *MoGLN3* had no effect on the vegetative growth of *M. oryzae*. However, Δ*Mogln2* was significantly reduced in growth on CM, SDC, and OTM. These findings echoed a previous study where glutamine synthetase *GlnA1* of *Mycobacterium tuberculosis* was required for growth in human THP-1 macrophages and guinea pigs [41]. The Δ*Mogln2* was attenuated in growth on MM medium, and supplementation of MM medium with l-glutamine rescued the growth defects, demonstrating that glutamine auxotroph in rice blast fungus occurs via inactivation of a single copy of GS (*MoGLN2*); this differs from a previous study with *Sinorhizobium meliloti* (formerly *Rhizobium meliloti*) that required all three GS genes to be inactivated to generate a strain that was auxotrophic for l-glutamine [73].

Intracellular glutamine test assays showed that glutamine levels were significantly reduced in Δ*Mogln2,* remained unchanged in Δ*Mogln1,* and were significantly higher in Δ*Mogln3*. An explanation for unchanged glutamine levels in the Δ*Mogln1* mutant might be that glutamine was being synthesized by *MoGLN2*. The higher glutamine levels in Δ*Mogln3* were attributed to up-regulation of both *MoGLN1* and *MoGLN2*. *MoGLN1* was previously reported to reduce higher glutamine turnovers in the Δ*asd4* mutant after the *MoGLN1* gene was deleted in the Δ*asd4* mutant background [57]. This shows that *MoGLN1* might be involved in glutamine biosynthesis. Therefore, the likely source of low intracellular glutamine detected in Δ*Mogln2* was from up-regulation of *MoGLN1*; however, *MoGLN1* cannot supplement *MoGLN2* to produce sufficient glutamine levels required for fungal development and pathogenicity. The amount of other amino acids was found to have significantly increased in Δ*Mogln2* and correlates with a study on *S. cerevisiae* where the intracellular amount of other amino acids was found to have increased in the *S. cerevisiae* Δ*Scgln1* mutant [66]. The increased levels of the other amino acids in Δ*Mogln2* might be attributed to enhanced biosynthesis of amino acids, as our RNA sequencing data revealed that genes related to amino acid biosynthesis, such as those involved in translation, amino acid activation, tRNA aminoacylation, tRNA aminoacylation for protein translation, amide biosynthetic process, and peptide biosynthetic process, were found to be up-regulated in Δ*Mogln2* mutants.

Like in many fungal pathogens, conidiation and appressorium development are key steps in the disease cycle of *M. oryzae*. Upon landing on the host surface, the conidia begin to produce germ tubes and eventually develop into a specialized infection structure called appressorium, with 8 MPa turgor pressure at the tips, which helps the fungus penetrate host cell barriers [39]. Analysis of the conidiation profiles of the three *MoGLN* mutants showed that both Δ*Mogln1* and Δ*Mogln3* had no effect on the asexual process in rice blast fungus. However, Δ*Mogln2* failed to produce conidia in the different sporulation media tested. This observation was consistent with stage-specific qRT-PCR results, which showed that *MoGLN2* was highly expressed at the conidiation stage. It is well shown that the aerial hyphal formation plays a crucial role during conidiophores differentiation and conidiation [74,75,76,77]. The Δ*Mogln2* exhibited hyphal autolysis, with poor development of aerial hyphal on sporulation media SDC, OTM, and rice bran, indicating that the conidiation defects of the mutant may be due to the inability to form conidiophores. Sporulation defects of the mutant catalyzing amino acid biosynthetic process could be remediated by adding corresponding exogenous amino acid [78,79]. In this study, several attempts were made to supplement different sporulation media with l-glutamine concentration of 1 mM, 2 mM, 5 mM, 10 mM, 20 mM, 40 mM, and 60 mM; however, conidiogenesis could still not be restored by Δ*Mogln2.* l-glutamine was reported to be unstable compared to the other amino acids in aqueous solution [80] and given the long incubation time required for activation of conidiation in *M. oryzae* on sporulation medium (at least 10 days in dark and three days in light), it is most likely that a substantial amount of the initial l-glutamine is degraded in the culture medium. This could be a possible reason why exogenous l-glutamine did not rescue conidiation defects in Δ*Mogln2.*

Glutamine has been reported as one of the amino acids required by fungal pathogens during host colonization for successful infection to occur. For instance, analysis of amino acid changes during sunflower infection by *Botrytis cinerea* showed glutamine derived from the host was required by the fungus during in planta infection [81]. Moreover, colonization of *Piriformospora indica* to its host during in planta infection required glutamine [82]. In this study both Δ*Mogln1* and Δ*Mogln3* had a sufficient amount of glutamine, and thus they were able to colonize the barley cells and cause infection. However, the Δ*Mogln2* mutant, which had low levels of intracellular glutamine, failed to form appressorium-like structures and thus was completely nonpathogenic on barley leaves. The failure of the Δ*Mogln2* mutant to cause infection on barley leaves echoed previous studies with *Mycobacterium species,* where loss of function of *GLN1A* and *GLN1* resulted in attenuated virulence on their respective hosts [41,83]. Our findings and previous studies on *Mycobacterium species* clearly demonstrate that glutamine biosynthesis mediated by glutamine synthetase is a critical process for pathogenic microorganisms to cause infection in their host.

The fungal cell wall is a vital structure with great plasticity and is crucial for maintaining cellular integrity and viability. The cell wall plays an important role in different functions, including controlling cellular permeability and cushioning the cell from osmotic and mechanical stress [84,85,86]. In addition, the cell wall facilitates smooth interactions of the cellular components with the external environment through adhesins and a large number of receptors; upon their activation, triggers signal transductions inside the cell [84]. Variations in nutrient availability result in changes in the expression of enzymes required for cell wall biosynthetic enzymes. It was previously reported that the loss of function of glutamine synthetase in *S. cerevisiae* resulted in Δ*Scgln1* mutant showing a defect in cell wall integrity [66]. Furthermore, glutamine synthetase *GLN1-A* in pathogenic *Mycobacterium bovis* was reported to be essential in cell wall resistance [83]. Similarly in this study, we established that the Δ*Mogln2* mutants displayed increased sensitivity to cell wall stressors when cultured on CM medium containing (CR, CFW, and SDC). Moreover, less protoplast was formed in the Δ*Mogln2* mutant compared to Guy11, Δ*Mogln1,* and Δ*Mogln3* after being treated with lytic enzyme, implying an altered cell wall structure and making it resistant to degradation by lytic enzymes. Mps1 phosphorylation has been used as a marker for cell wall integrity tests [65]. In this study, the phosphorylation level of MoMps1 was decreased in Δ*Mogln2* but not Δ*Mogln1* and Δ*Mogln3*, further indicating the involvement of *MoGLN2* in cell wall fungal cell integrity. Our findings on the contribution of *MoGLN2* in the maintenance of cell wall integrity in rice blast fungus and previous studies on *GLN1* of *S. cerevisiae* and *GLN1-A* in pathogenic *Mycobacterium bovis* demonstrate the important role of glutamine synthetase in cell wall integrity in living organisms.

It has been well demonstrated that ROS homeostasis is essential for fungal development [87,88]. In rice blast fungus, loss of *MoSEC22* resulted in reduced intracellular ROS levels, with the mutant being highly sensitive to H_2_O_2_ and losing its virulence [89]. In this study, deletion of Δ*Mogln2* was highly sensitive both on 2 mM and 5 mM H_2_O_2_, indicating that *MoGLN2* likely plays an important role in oxidative stress tolerance in rice blast fungus.

In summary, we established that among the three *MoGLN* genes, *MoGLN2* is required for glutamine biosynthesis and is essential for growth, conidiogenesis, appressorium formation, and pathogenicity. Moreover, we confirmed that *MoGLN2* is involved in the maintenance of cell wall integrity and oxidative stress tolerance in rice blast fungus. These findings provide an attractive target for the development of antifungal agents required to control the devastating effects of plant fungal pathogens.

## Figures and Tables

**Table 1:** Intracellular free amino acids.

Amino Acid	*p*-Value	Guy11		Δ*Mogln2*	
		Mean		Mean	
		(μg/g) SD		(μg/g) SD	
Alanine	0.004	2158.11	41.42232	3521.255	111.47538
Serine	0.015	394.285	88.46613	911.845	13.73908
Proline	0.01	234.075	22.90319	1054.775	116.4817
Valine	0.008	399.47	1.24451	1125.905	90.12076
Isoleucine	0.014	150.165	24.71338	604.355	73.88559
Threonine	0.03	412.59	9.48937	2922.265	405.98536
Aspartate	0.097	287.76	51.36424	503.8	89.35001
Leucine	0	219.82	3.73352	880.38	7.43876
Asparagine	0.012	119.015	35.65939	421.79	31.09856
Lysine	0.038	2444.65	479.2487	17,505.03	4272.45938
Glutamate	0.024	1731.585	84.53462	4348.05	583.39138
Methionine	0.026	50.34	3.6911	186.5	31.72081
Histidine	0.094	474.54	36.44428	1734.325	585.7319
Phenylalanine	0.007	4.1295	4.1295	460.365	38.33226
Arginine	0.282	2692.36	310.1512	4231.13	1456.97938
Tryptophan	0.002	50.755	0.3182	241.79	12.37437
Tyrosine	0.064	271.06	60.99503	1841.21	589.82605

SD represents standard deviation; mean is the average of two independent replicates.

## Data Availability

Not applicable.

## Supplementary Materials

The following are available online at https://www.mdpi.com/article/10.3390/jof7060463/s1, Figure S1, Heatmap representation of differentially expressed genes upon deletion of *MoGLN2*; Figure S2, KEGG Enrichment for up-regulated and down-regulated pathways in Δ*Mogln2* muta*nt*: (**A**) KEGG enrichment for up-regulated genes (**B**) KEGG enrichment for down-regulated genes; Table S1, Primers used in this study.
