# Supplementary material for: MoGLN2 Is Important for Vegetative Growth, Conidiogenesis, Maintenance of Cell Wall Integrity and Pathogenesis of Magnaporthe oryzae
Source: J Fungi (Basel). 2021 Jun 8;7(6):463. doi: 10.3390/jof7060463 (PMC8229676; doi:10.3390/jof7060463)
Supplement: Supplementary file 1 [file jof-07-00463-s001.zip › jof-1225823-supplementary/Figure S2.pdf]

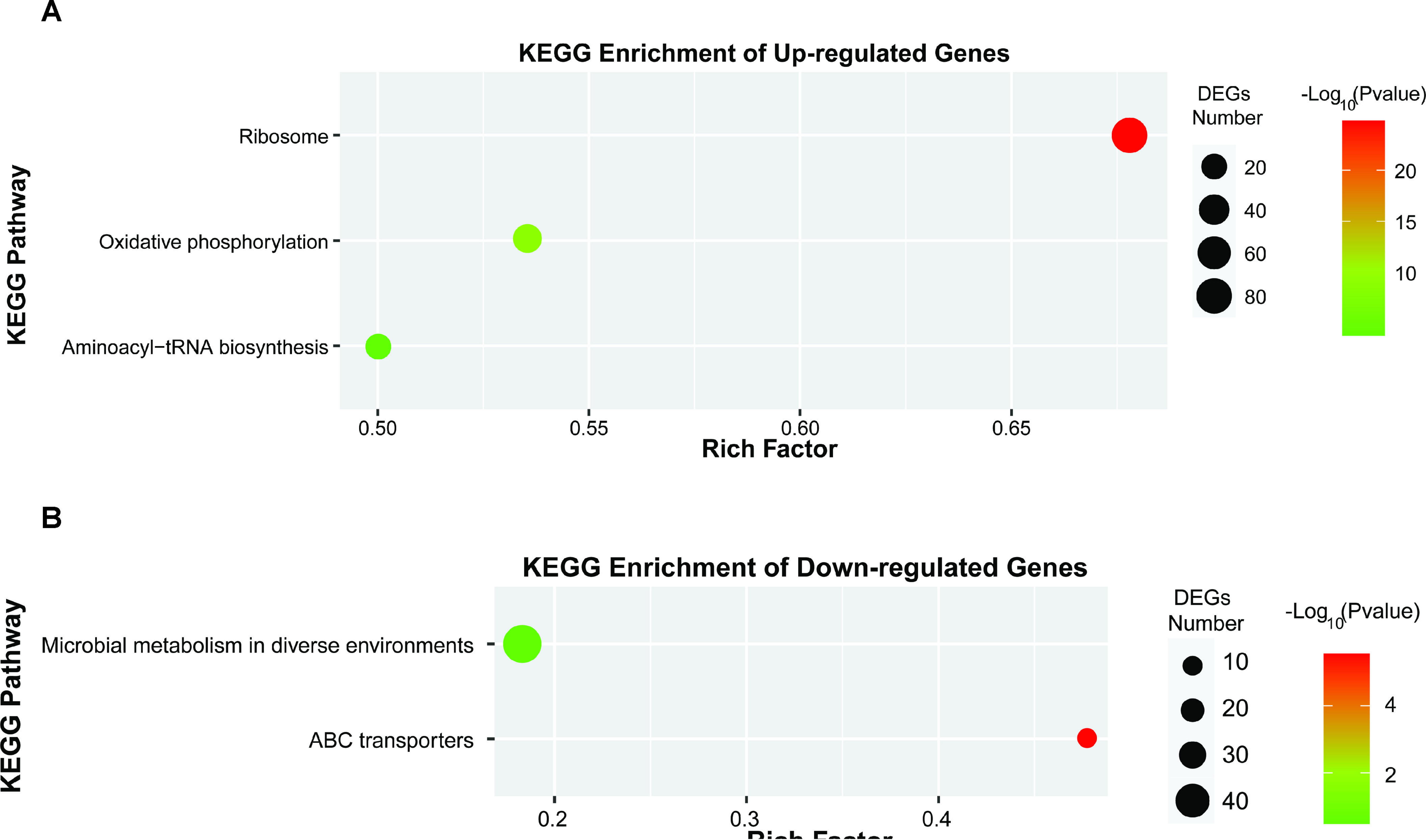
A
KEGG Enrichment of Up-regulated Genes
KEGG Pathway
Ribosome
Oxidative phosphorylation
Aminoacyl−tRNA biosynthesis
0.50
0.55
0.60
0.65
Rich Factor
DEGs Number
20
40
60
80
-Log10(Pvalue)
20
15
10
B
KEGG Enrichment of Down-regulated Genes
KEGG Pathway
Microbial metabolism in diverse environments
ABC transporters
0.2
0.3
0.4
DEGs Number
10
20
30
40
-Log10(Pvalue)
4
2
