# Supplementary material for: MoGLN2 Is Important for Vegetative Growth, Conidiogenesis, Maintenance of Cell Wall Integrity and Pathogenesis of Magnaporthe oryzae
Source: J Fungi (Basel). 2021 Jun 8;7(6):463. doi: 10.3390/jof7060463 (PMC8229676; doi:10.3390/jof7060463)
Supplement: Supplementary file 1 [file jof-07-00463-s001.zip › jof-1225823-supplementary/Table S1.pdf]

Table S1 Primes used in this study

Primer name	Sequence (5-3′)	Remarks
GLN1 AF	GAACAAAAGCTGGGTTGTGGAGCCCAACGTTAC	Amplify 5′*MoGLN1* fragment
GLN1 AR	CAGCGGCGCGCCGAAAATATGGAGCGAGGAGGAT	Amplify 5′*MoGLN1* fragment
GLN1 OF	GCAGAGTACGTCTGGATTG	Amplify *MoGLN1* ORF
GLN1 OR	GCAGGTGACGGCATAATTC	Amplify *MoGLN1* ORF
GLN1 BF	ACCGGGCCGGCCGGATGAGGTTGCCGTAGGACATC	Amplify 3′*MoGLN1* fragment
GLN1 BR	GGTGGCGGCCGCTCTGCGTGGTCAATATGGCGAAC	Amplify 3′*MoGLN1* fragment
HPH F	TGGAGCTAGTGGAGGTCAAC	Amplify HPH probe
HPHR	CGGCCGCTCTAGAACTAGTG	Amplify HPH probe
HYG/F	GGCTTGGCTGGAGCTAGTGGAGGTCAA	Amplify 5′*MoGLN1* fragment
HY/R	GTATTGACCGATTCCTTGCGGTCCGAA	Amplify 5′*MoGLN1* fragment
YG/F	GATGTAGGAGGGCGTGGATATGTCCT	Amplify 3′HPH fragment
HYG/R	AACCCGCGGTCGGCATCTACTCTATTC	Amplify 3′HPH fragment
GLN2 AF	GAACAAAAGCTGGGTGCTAACAGGGCCAGGTAAAG	Amplify 5′*MoGLN2* fragment
GLN2 AR	CAGCGGCGCGCCGAAGATTTGACTGGCGGCATTTC	Amplify 5′*MoGLN2* fragment
GLN2 OF	TGTCACATACGTCGAGAACC	Amplify *MoGLN2* ORF
GLN2 OR	ACCGACACCACAGTAGTATG	Amplify *MoGLN2* ORF
GLN2 BF	ACCGGGCCGGCCGGAAAACCTTGGCTTGGCGAACC	Amplify 3′*MoGLN2* fragment
GLN2BR	GGTGGCGGCCGCTCTCGCGCAACTTGACTTTGTCC	Amplify 3′*MoGLN2* fragment
GLN3AF	GAACAAAAGCTGGGTCAGGAGGTGTGTGTGGAATG	Amplify 5′*MoGLN3* fragment
GLN3AR	CAGCGGCGCGCCGAAACTTCTCTGTTCAGGCAAAGC	Amplify 5′*MoGLN3* fragment
GLN3 OF	CCAAGGATGGCTTTGGTTTC	Amplify *MoGLN3* ORF
GLN3 OR	TGGCCTCGTGAGACTGTA	Amplify *MoGLN3* ORF
GLN3 BF	GACAATCGGCTGCTCTGATGCCAGACTTGCATATAGGG	Amplify 3′*MoGLN2* fragment
GLN3 BR	CTCCTATGAGTCGTTTACCCAAATAGGGAAGGGCTGGG	Amplify 3′*MoGLN2* fragment
QGLN1 FW	ATGGCGACACAACAGGTATC	qPCR of *MoGLN1*
QGLN1 RV	CTGTCAATCCAGACGTACTC	qPCR of *MoGLN1*
QGLN3 FW	ACATGCACGACCAGACCTAC	qPCR of *MoGLN3*
QGLN3 RV	AAGAACGGGATGTCGTTCTC	qPCR of *MoGLN3*
Buf1 F	ACGCCGTCTACTCAGGATCA	qPCR of *MoBUF1*
Buf1 R	TCTCGCCGTTTGGAATGTAT	qPCR of *MoBUF1*
Alb F	GCAATGTCGGTCCCAACTAC	qPCR of *MoALB1*
Alb R	ATCTCAAAGGCGATGACACC	qPCR of *MoALB1*
Rsy1 F	CGACTCCAAGGACTGGGATA	qPCR of *MoRSY1*
Rsy1 R	GTCCTCGGACACCTTCTCC	qPCR of *MoRSY1*
QCOS1 F	TGTGGTTCCTGTCCCTTCTC	qPCR of *MoCOS1*
QCOS1 R	TGTAGCCTGGCGAGTCCAAC	qPCR of *MoCOS1*
QCON6 F	AGTCCAAGCAGCACTCGAAG	qPCR of *MoCON6*
QCON6 R	CTTGATGGCAGCCTTGAGAC	qPCR of *MoCON6*
QCON7 F	CACCACCAGCTTTCCTCATC	qPCR of *MoCON7*
QCON7 R	AACCTGACTGCGAGTAATCC	qPCR of *MoCON7*
QHOX6 F	TTCCCTTCCCAGTTCACTTC	qPCR of *MoHOX6*
QHOX6 R	GCTGCTACCGGGAGAATTTG	qPCR of *MoHOX6*
QMOACTIN F	CCATGTACCCTGGTCTTTCG	qPCR of *MoACTIN*
QMOACTIN R	TTCGAGATCCACATCTGCTG	qPCR of *MoACTIN*
COM1 F	TGAGCGATGACATGCTTGAG	qPCR of *MoCOM1*
COM1 R	GAGTGATGGGTGCTGTGTTG	qPCR of *MoCOM1*
QSTUA F	AGGACACGACATGTACTACC	qPCR of *MoSTUA*
QSTUA R	CTGAGGAGGAGCTGGATAAG	qPCR of *MoSTUA*
QGLN2 F	TGTCACATACGTCGAGAACC	qPCR of *MoGLN2*

QGLN2 R	CAGACGTACTCTGCGATGAC	qPCR of *MoGLN2*
GLN3COMF	GAACAAAAGCTGGGT3'GGTACCCGCTTTCCTCCGCCATTTAG	Amplify *MoGLN3* Comp frag
GLN3COM R	CTGCAGGCATGCAAGAAGCTTAACTGTTTCGATGTACCTCTT	Amplify *MoGLN2* Comp frag
GLN2 COM F	GAACAAAAGCTGGGT3'TCCTCCTGTCGGTGAGTTCTG	Amplify *MoGLN2* Comp frag
GLN2 COM R	CTGCAGGCATGCAAGTGCCTCGCCGAAGCAGGTC	Amplify *MoGLN2* Comp frag
GLN1 COMF	GAACAAAAGCTGGGT3'TGAGGTTGCCGTAGGACATC	Amplify *MoGLN1* Comp frag
GLN1 COMR	CTGCAGGCATGCAAGTCTGCGTGGTCAATATGGCGAAC	Amplify *MoGLN1* Comp frag
