# Supplementary figures and images for: MoGLN2 Is Important for Vegetative Growth, Conidiogenesis, Maintenance of Cell Wall Integrity and Pathogenesis of Magnaporthe oryzae
Source: J Fungi (Basel). 2021 Jun 8;7(6):463. doi: 10.3390/jof7060463 (PMC8229676; doi:10.3390/jof7060463)

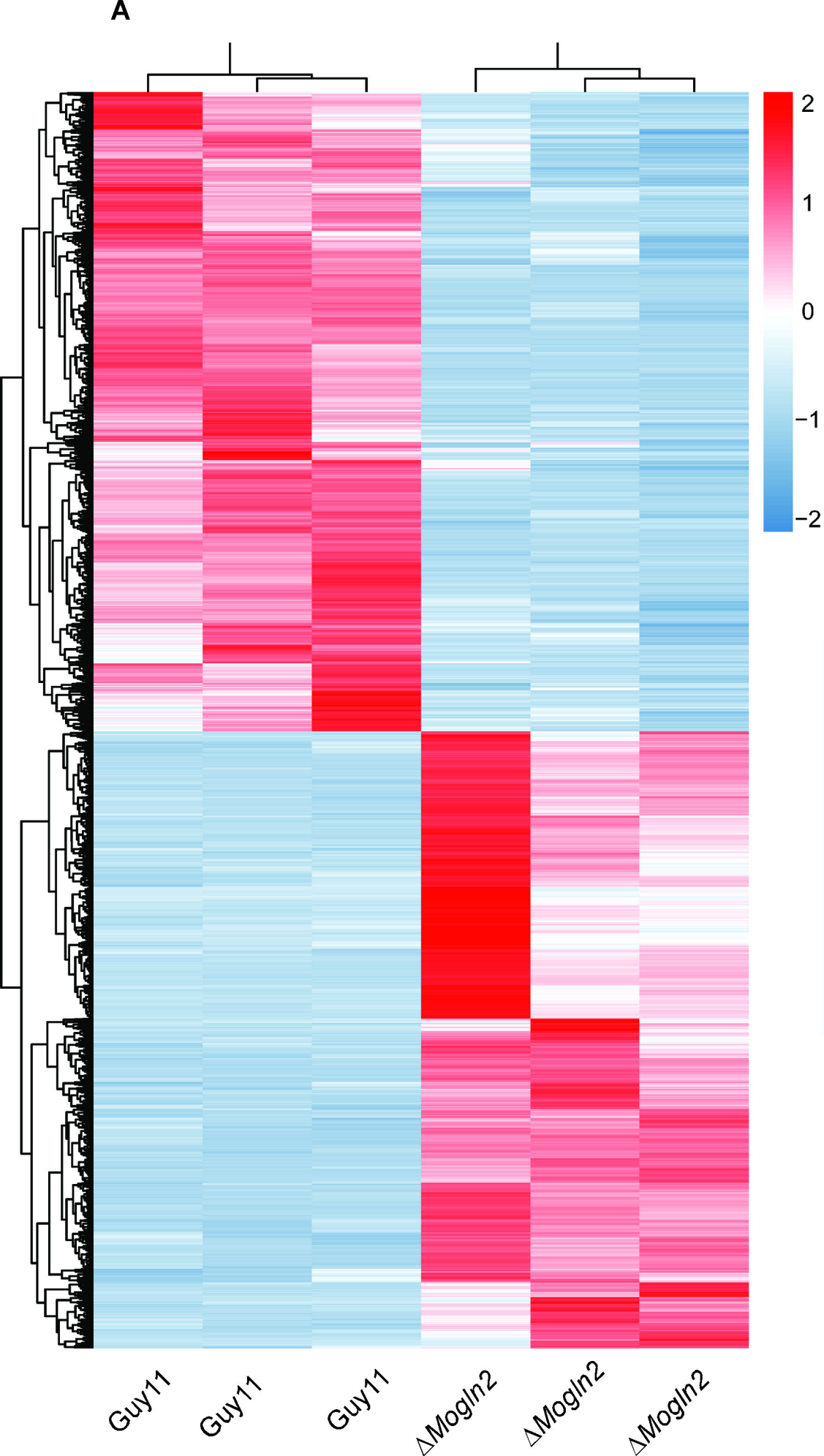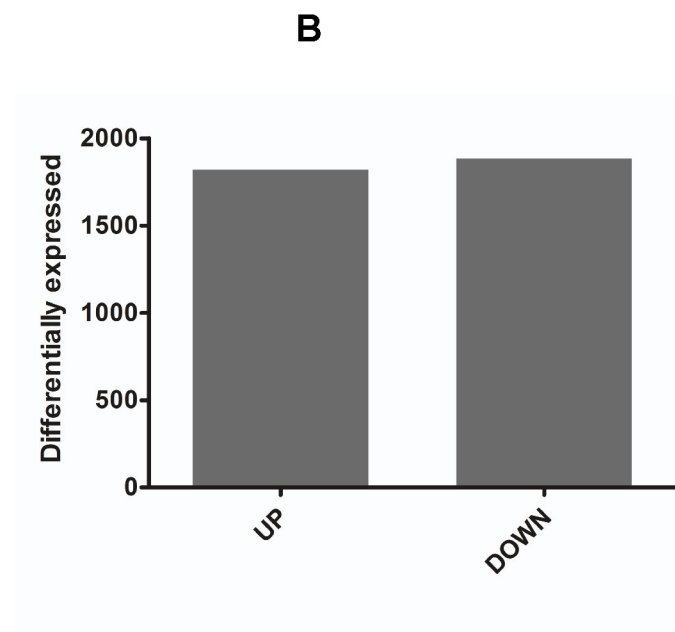

Supplement: Supplementary file 1 [file jof-07-00463-s001.zip › jof-1225823-supplementary/Figure S1.pdf]
